# Identification of a novel *EVC* variant in a Han‐Chinese family with Ellis‐van Creveld syndrome

**DOI:** 10.1002/mgg3.885

**Published:** 2019-07-23

**Authors:** Xiangjun Huang, Yi Guo, Hongbo Xu, Zhijian Yang, Xiong Deng, Hao Deng, Lamei Yuan

**Affiliations:** ^1^ Department of General Surgery The First Affiliated Hospital of Hunan University of Chinese Medicine Changsha China; ^2^ Department of Medical Information, School of Life Sciences Central South University Changsha China; ^3^ Center for Experimental Medicine The Third Xiangya Hospital, Central South University Changsha China

**Keywords:** dysplasia, Ellis‐van Creveld syndrome, *EVC*, nonsense variant

## Abstract

**Background:**

Ellis‐van Creveld syndrome (EVC), a very rare genetic skeletal dysplasia, is clinically characterized by a tetrad consisting of chondrodystrophy, polydactyly, ectodermal dysplasia, and cardiac anomalies. The aim of this study was to identify the genetic defect for EVC in a five‐generation consanguineous Han‐Chinese pedigree.

**Methods:**

A five‐generation, 12‐member Han‐Chinese pedigree was enrolled in this study. Exome sequencing was applied in the proband to screen potential genetic variant(s), and then Sanger sequencing was used to identify the variant in family members and 200 unrelated ethnicity‐matched controls.

**Results:**

A novel homozygous variant, c.2014C>T, p.(Q672*), in the EvC ciliary complex subunit 1 gene (*EVC*), was detected in the patient, which was cosegregated with the disease in the family and absent in the controls.

**Conclusion:**

The identified novel homozygous *EVC* variant, c.2014C>T, p.(Q672*), was responsible for EVC in this Han‐Chinese pedigree. The findings in this study extend the *EVC* mutation spectrum and may provide new insights into EVC causation and diagnosis with implications for genetic counseling and clinical management.

## 1 INTRODUCTION

Ellis‐van Creveld syndrome (EVC, OMIM 225500), also called “chondroectodermal dysplasia” and “mesoectodermal dysplasia”, is an extremely uncommon genetic skeletal disorder inherited in an autosomal recessive pattern, clinically characterized by a tetrad of chondrodystrophy, polydactyly, ectodermal dysplasia, and congenital heart defects (Fischer et al., 2015; Hao, Fan, He, Liu, & Ge, 2016; Naqash, Alshahrani, & Simasetha, 2018). It was initially summarized by Ellis and van Creveld in 1940 (Shaik, Raviraj, Dirasantchu, & Venkata, 2016). As one of the rarest ciliopathies, its precise prevalence is unknown but is estimated to have a low prevalence of 7/1,000,000 in the general population. It has been reported as most prevalent in Amish, Brazilian, Ashkenazi Jewish, and Arab populations due to consanguinity (Al‐Fardan & Al‐Qattan, 2017; Chen et al., 2010; Shaik et al., 2016). EVC patients may have abnormal skeletal and extraskeletal manifestations, including disproportionate short‐limb dwarfism, narrow chest, cubitus valgus, genu valgus, postaxial polydactyly, sparse hair, multiple oral frenula, nail and teeth dysplasia, and congenital heart defects (Chen et al., 2010; Fischer et al., 2015; Ibarra‐Ramirez et al., 2017; Rao, Sahu, Kareem, Devasia, & Shetty, 2017; Valencia et al., 2009). Neither cognitive nor motor development is affected (Fischer et al., 2015). The most severe EVC phenotype is cardiorespiratory complication. Nearly, half of all EVC patients die during childhood though remedial heart surgery may raise survival rates (Nguyen et al., 2016; Pérez‐Andreu, Ray, Arribas, & Sánchez, 2015). Histopathologic features include disarranged chondrocytes in long bone metaphysis and, to a lesser extent, in central vertebrae metaphysis (Lichiardopol & Militaru, 2006).

In EVC, homozygous or compound heterozygous mutations of the EvC ciliary complex subunit 1 gene (*EVC*, OMIM 604831) or the EvC ciliary complex subunit 2 gene (*EVC2*, OMIM 607261) are accused to be the culprits. These two genes lie head‐to‐head on chromosome 4p16, encoding proteins present in the primary cilium basal body and functioning in the same biological pathway (Al‐Fardan & Al‐Qattan, 2017; Shi, Luo, Ahmed, Attaie, & Ye, 2016; Umair et al., 2017). Nonsense and frameshift mutations are responsible for 70% of all pathogenic mutations in these two genes. They express in the developing skeleton, heart, kidneys, and lungs (Aziz, Raza, Ali, & Ahmad, 2016; Shi et al., 2016).

This study sought to reveal the genetic factor giving rise to EVC in a five‐generation consanguineous Han‐Chinese family. A novel homozygous *EVC* c.2014C>T variant, which may result in a protein truncation (p.(Q672*)) and a loss of protein function, was identified. These may hinder normal hedgehog signaling pathway, and disrupt normal endochondral growth, and orofacial, limb, and skeletal development and maintenance (Chen, Chen, Chern, Su, & Wang, 2012; Ibarra‐Ramirez et al., 2017; Ruiz‐Perez & Goodship, 2009; Valencia et al., 2009).

## 2 MATERIALS AND METHODS

### 2.1 Ethical compliance

Written informed consent was obtained from participants or the legal guardian. This study had received approval from the Institutional Review Board of the Third Xiangya Hospital, Central South University, Changsha, China.

### 2.2 Participators and clinical examination

**Figure 1:**
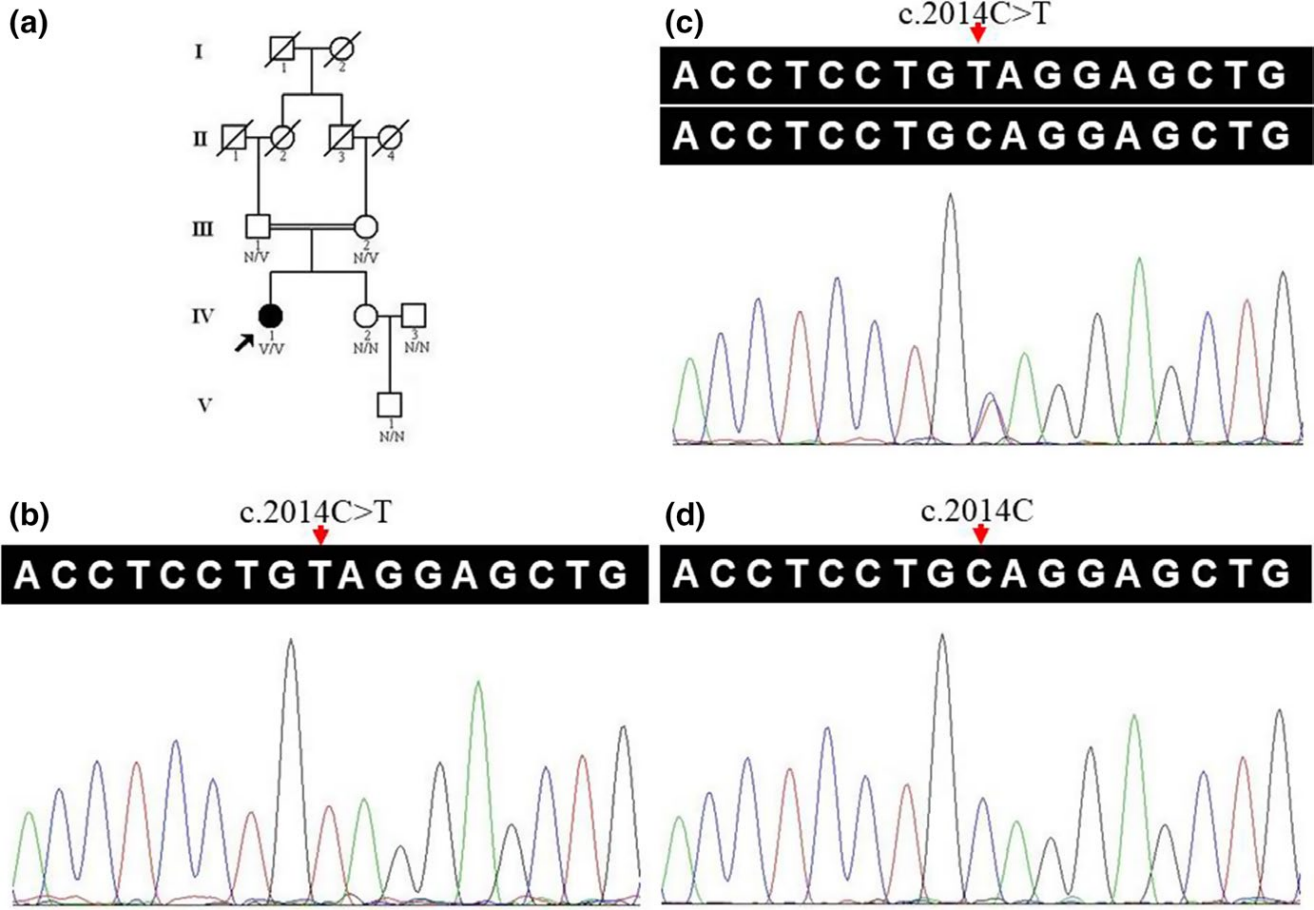
The c.2014C>T variant in the *EVC* (NM_153717.3) in a pedigree with EVC. (a) Pedigree of the family with autosomal recessive EVC. Double lines indicate the consanguineous marriage. Square denotes male family member, circle presents female family member, slashed symbol indicates deceased family member, fully shaded symbol shows patient with EVC, and open symbol presents EVC‐free member. N, the *EVC* wild‐type allele; V, the allele with *EVC* c.2014C>T variant. (b) The *EVC* sequence with homozygous c.2014C>T variant (IV:1). (c) The *EVC* sequence with heterozygous c.2014C>T variant (III:2). (d) The normal *EVC* sequence of EVC‐free member (IV:2). *EVC*, the EvC ciliary complex subunit 1 gene; EVC, Ellis‐van Creveld syndrome

A five‐generation Han‐Chinese family with EVC was recruited at the Third Xiangya Hospital, Central South University, Changsha, China (Figure 1a). Six individuals, including the proband (IV:1), the unaffected first‐cousin parents (III:1 and III:2), and three other unaffected family members (IV:2, IV:3, and V:1) were involved in this study, and blood samples were collected. Detailed clinical medical histories and physical examinations were collected from all participants. Blood specimens were also collected from 200 unrelated, ethnically matched controls (male/female: 100/100, age 33.4 ± 6.9 years) which lacked EVC diagnostic features. Each participant underwent thorough examination for clinical diagnosis or exclusion, including general examination such as polydactyly and dysplastic nails, oral check‐up such as oral frenula, and teeth, limbs, and cardiovascular inspection.

### 2.3 Exome capture

Genomic DNA (gDNA) was separated from peripheral blood lymphocytes via a phenol–chloroform extraction (Huang et al., 2018). Proband (IV:1) exome sequencing was performed for unveiling genetic causation of EVC in this pedigree, following the established protocol of BGI‐Shenzhen (Fan et al., 2019; Yuan et al., 2016). A paired‐end DNA library was constructed and whole‐exome capture was performed utilizing BGI exome V4 kit. The qualified circular DNA library was sequenced on a BGISEQ‐500 platform (Fan et al., 2019).

### 2.4 Read mapping and variant analysis

The human reference genome was accessed from UCSC Genome Browser (Human GRCh37/hg19). Sequencing data were aligned using Burrows–Wheeler Aligner and SOAPaligner (v2.21). SOAPsnp (v1.05), Genome Analysis Toolkit HaplotypeCaller, Platypus, and SAMtools were applied for calling single nucleotide polymorphisms (SNPs) and insertions/deletions (indels), after duplicate reads, primarily produced by polymerase chain reaction (PCR) amplification, were deleted. Variant Effect Predictor was used to annotate the identified variants (Huang et al., 2018; Wang et al., 2019). Common variants or nonpathogenic variants were filtered out using the human genetic variation databases, including the Single Nucleotide Polymorphism database (dbSNP build 138), 1000 Genomes Project, and HapMap project. Variants were further annotated using the BGI in‐house exome databases with 2,375 Chinese controls, as well as the NHLBI Exome Sequencing Project (ESP) 6500 (https://evs.gs.washington.edu/EVS/) and Exome Aggregation Consortium (ExAC, https://exac.broadinstitute.org/) databases. Bioinformatics tools including Polymorphism Phenotyping version 2 (PolyPhen‐2, http://genetics.bwh.harvard.edu/pph2/), Protein Variation Effect Analyzer (PROVEAN, http://provean.jcvi.org/), and MutationTaster (http://www.mutationtaster.org/) were used to evaluate whether amino acid substitutions affected protein structures and functions (Fan et al., 2019; Hu et al., 2017). PCR amplification was conducted with the gDNA samples and the designed locus‐specific primers, and the presence of potential causative variant was further tested by Sanger sequencing on the Applied Biosystems 3500 genetic analyzer (Applied Biosystems Inc.) (Guo et al., 2013). The primer sequences were as follows: 5′‐TGCTTTCCTCAAAGGTCCAC‐3′ and 5′‐GCCACCATGCTGGTCTTAC‐3′. According to guidelines for variant interpretation of American College of Medical Genetics and Genomics (ACMG), the detected variant was further categorized (Richards et al., 2015).

## 3 RESULTS

### 3.1 Clinical findings

**Figure 2:**
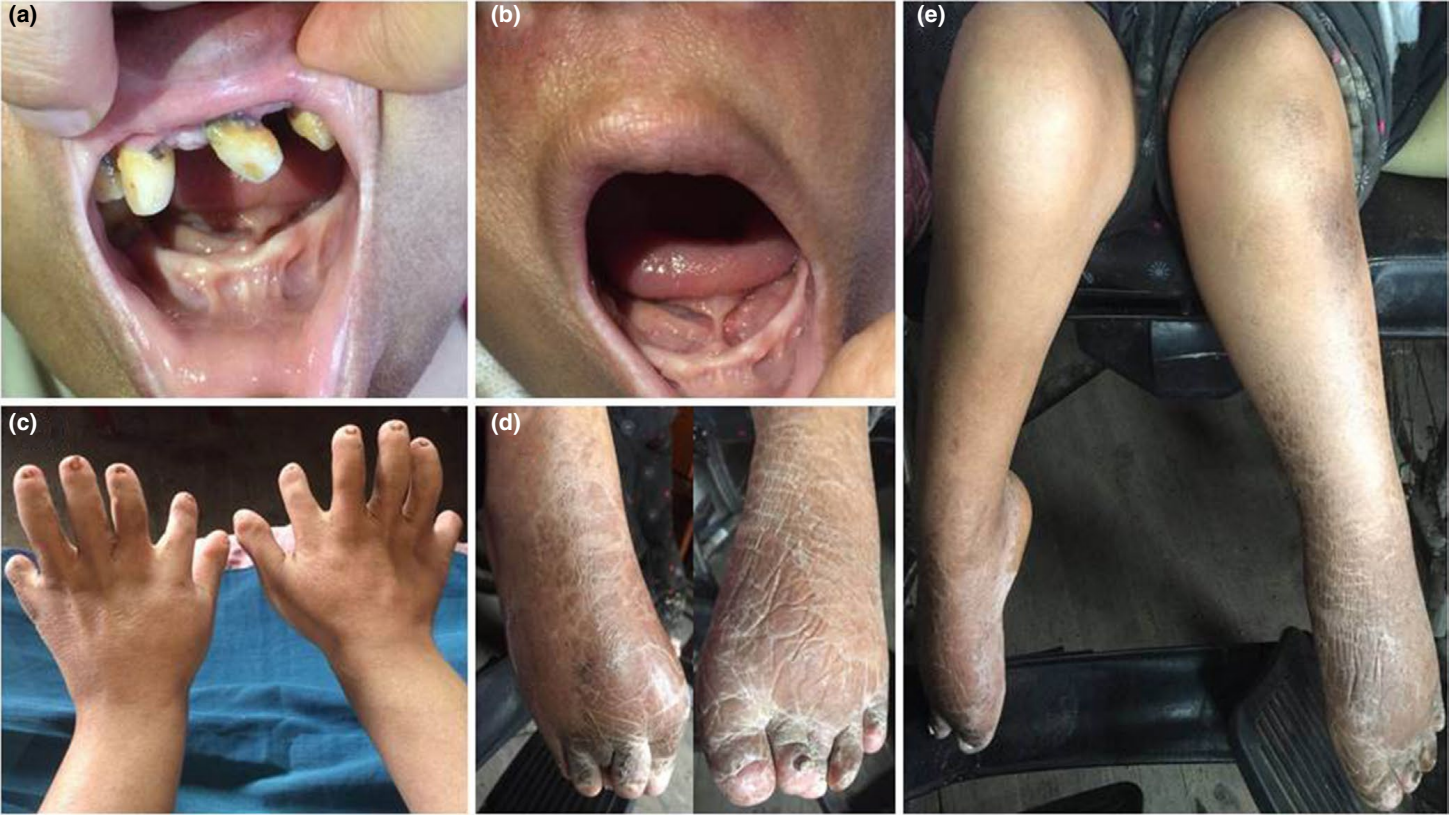
Clinical features of patient with EVC. (a) Absence of maxillary incisors, mandibular anodontia, and alveolar ridge defect. (b) Multiple oral frenula. (c, d) Bilateral ulnar polydactyly, hypoplasia of the distal phalanges, and dysplastic nails. (e) Genu valgus. EVC, Ellis‐van Creveld syndrome

The EVC diagnosis was made using characteristic clinical manifestations. The proband (IV:1) was a 42‐year‐old female, born to an unaffected consanguineous couple. Physical examination revealed disproportionate dwarfism, bilateral ulnar polydactyly, distal phalanges hypoplasia, dysplastic nails, and genu valgus. Intraoral examination showed multiple oral frenula, absence of maxillary incisors, mandibular anodontia, and alveolar ridge defect (Figure 2). No congenital heart defect was found. All relatives were unaffected with no similar features.

### 3.2 Genetic analysis

A total of 38,706.61 Mb mapped to reference genome were generated by exome sequencing on gDNA from the proband (IV:1), with a coverage of 99.93% in the target region. The mean sequencing depth of target region was 277.04‐fold, and 99.17% of the target region was covered by at least 10‐fold. After a prioritized filtering process for determining the potential pathogenic variant (Fan et al., 2019; Xiao et al., 2018), a homozygous nonsense variant in the *EVC*, a known EVC‐causing gene, was remained to be validated.

After the Sanger sequencing and filtering via the Human Gene Mutation Database (HGMD, http://www.hgmd.cf.ac.uk/), a novel homozygous variant, c.2014C>T, p.(Q672*), in exon 14 of the *EVC* (NM_153717.3), was confirmed in the proband (Figure 1b). Sanger sequencing showed the variant heterozygosity in her unaffected first‐cousin parents (Figure 1c). This variant was not present in three other unaffected family members (IV:2, IV:3, and V:1, Figure 1d), BGI in‐house exome databases, NHLBI ESP6500, or 200 unrelated controls. No homozygote (0/117,630) was recorded in the ExAC database. The homozygous variant cosegregated with the disease in this family, suggesting it is likely to be the pathogenic variant. PROVEAN predicted it was deleterious, and MutationTaster revealed it was “disease causing” (classification due to nonsense‐mediated mRNA decay, NMD), further supporting the identified *EVC* variant was deleterious for EVC. The variant is classified as “pathogenic” following the ACMG variant interpretation guidelines.

## 4 DISCUSSION

This study identified, a novel homozygous variant, c.2014C>T, p.(Q672*), in the *EVC* present in a consanguineous Han‐Chinese family with EVC. The patient manifested typical EVC clinical features, including disproportionate dwarfism, multiple oral frenula, absence of maxillary incisors, mandibular anodontia and alveolar ridge defect, bilateral ulnar polydactyly, distal phalanges hypoplasia, dysplastic nails, and genu valgus, but no congenital heart defect. Various symptoms were evidenced in early onset and a special attention was needed from birth. The homozygous variant cosegregated with the disorder in this family, and was absent in the 200 controls, public, and in‐house exome databases, suggesting that it is pathogenic. Computer‐based analysis further confirmed the predicted deleterious effect of the variant.

The *EVC*, located at chromosome 4p16.2, contains 21 exons and encodes a 992‐amino acid protein (Ruiz‐Perez et al., 2000; Shi et al., 2016). It orients head‐to‐head with its homolog gene *EVC2*, which also encodes a single‐pass type I transmembrane protein (Vona et al., 2018). These two proteins interact directly to form a complex at the primary cilium membrane (EvC zone), which is essential for normal endochondral growth and intramembranous ossification (Shi et al., 2016; Umair et al., 2017).

At least 81 *EVC* mutations, in the homozygous or compound heterozygous state, have been reported as responsible for EVC in the HGMD and in published literature, including 25 missense/nonsense mutations, 19 splicing mutations, 14 small deletions, 9 small insertions, 1 small indel, 8 gross deletions, 2 gross insertions, and 3 complex rearrangements (D'Ambrosio et al., 2015; D'Asdia et al., 2013; Liu et al., 2018; Shi et al., 2016; Tompson et al., 2007). The common mutations usually produce premature termination codons, either directly or following a reading frame shift, and are thus predicted to lead to nonsense‐mediated decay of transcripts or nonfunctional truncated proteins (D'Asdia et al., 2013; Ibarra‐Ramirez et al., 2017; Valencia et al., 2009).

Weyers acrofacial dysostosis (WAD, OMIM 193530) and EVC are allelic disorders. WAD is a mild EVC phenotype caused by *EVC* or *EVC2* heterozygous mutations, and the last exon of *EVC2* may be a hot region for mutations (D'Asdia et al., 2013; Nguyen et al., 2016). WAD is distinguished from EVC by its inheritance mode and phenotypic severity, which does not have narrow chest or congenital cardiac anomalies (D'Asdia et al., 2013; Shi et al., 2016). The pathogenic mechanism of these two disorders is a cilia‐mediated, diminished response to hedgehog ligands (Ruiz‐Perez & Goodship, 2009). In EVC, biallelic nonsense and/or frameshift mutations in the *EVC* or *EVC2* are likely to result in NMD or truncated proteins, and thus lead to EVC in a loss‐of‐function mechanism. In WAD, mutations in the last exon of *EVC2* may escape NMD and the resulted, more stable EVC2 proteins may have a negative effect by gaining a function or affecting the normal protein (Ibarra‐Ramirez et al., 2017; Shen, Han, Zhang, Zhao, & Feng, 2011; Shi et al., 2016; Valencia et al., 2009).

*Evc* ^−/−^ mice represent a precise EVC model, presenting short limbs, short ribs, and dental abnormalities, but no polydactyly (Ruiz‐Perez et al., 2007). Abnormal epiphyseal and periosteal induction was observed in histology of cartilage plate growth, similar to the defects in Indian hedgehog signaling (Blair et al., 2011). *Evc* ^+/−^ mice have no obvious abnormalities (Ruiz‐Perez et al., 2007). Genetic animal studies further establish a loss‐of‐function mechanism in EVC progress.

In summary, a novel homozygous nonsense alteration, c.2014C>T, p.(Q672*), is likely the responsible variant for the EVC present in this Han‐Chinese family. The findings extend the *EVC* genotypic spectrum and have genetic counseling and clinical management implications. Due to the limited number of patients in this study and relative rarity of this disorder, more cases in additional families confirmed by genetic analysis, as well as functional evidence may strengthen the variant pathogenicity. Further and more extensive studies involving either, or both, in vitro and in vivo models with genetic deficiencies, as well as detection of disease‐causing mutations, should help reveal pathogenic mechanisms and assist in developing targeted *EVC* gene therapeutic strategies.

## CONFLICT OF INTEREST

The authors declare no conflict of interest.
